# Reliability and Construct Validity of a Mobile Application for the Finger Tapping Test Evaluation in People with Multiple Sclerosis

**DOI:** 10.3390/brainsci14040407

**Published:** 2024-04-21

**Authors:** Víctor Navarro-López, Roberto Cano-de-la-Cuerda, Pilar Fernández-González, Selena Marcos-Antón, Aitor Blázquez-Fernández, María Fernández-Cañas, Diego Fernández-Vázquez

**Affiliations:** 1 Department of Physical Therapy, Occupational Therapy, Rehabilitation and Physical Medicine, Faculty of Health Sciences, Rey Juan Carlos University, Alcorcón, 28922 Madrid, Spain; victor.navarro@urjc.es (V.N.-L.); diego.fernandez@urjc.es (D.F.-V.); 2 Motion Analysis, Biomechanics, Ergonomy and Motor Control Laboratory (LAMBECOM), Faculty of Health Sciences, Rey Juan Carlos University, Alcorcón, 28922 Madrid, Spain; 3 Multiple Sclerosis Association of Leganés (ALEM), Leganés, 28915 Madrid, Spain; selena.marcos@urjc.es (S.M.-A.); aitorblazquezfernandez@outlook.es (A.B.-F.); mariacaass@gmail.com (M.F.-C.)

**Keywords:** finger tapping test, mobile applications, multiple sclerosis, neurologic examination, upper extremity

## Abstract

The finger tapping test (FTT) is a tool to evaluate the motor performance of the hand and fingers and eye-hand coordination with applicability in people with multiple sclerosis (pwMS). The aim of this study was to evaluate the intra- and inter-rater reliability of the TappingPro^®^ mobile app and the construct validity between this app and validated clinical scales for motor performance in healthy subjects and pwMS. 42 healthy subjects (mean age 25.05) and 13 pwMS (mean age 51.69, EDSS between 3 and 7.5E) participated. FTT was performed with the TappingPro^®^ mobile app. All participants were examined twice, with a one-week interval between the two appointments. For the analysis of construct validity, the Jamar^®^ hydraulic hand dynamometer, Box and Blocks Test (BBT), and Nine Hole Peg Test (NHPT) were used. The intra-rater reliability showed a good correlation (Intraclass Correlation Coefficient, ICC > 0.787) for both upper limbs and both times of FTT for healthy subjects, and an excellent correlation (ICC > 0.956) for upper limbs and both times of FTT for pwMS. The ICC for the inter-rater reliability was good (ICC = 0.869) for the non-dominant upper limb in the FTT 10 s of the healthy subjects, and excellent (ICC > 0.904) for all the other measurements of the healthy subjects and pwMS. However, the Bland–Altman plots showed disagreement between observers and measurements that should be considered in the interpretation of clinical evaluations. The correlation analysis for healthy subjects showed poor associations between all variables, except for the association between hand grip strength and the FTT 60 s in the non-dominant upper limb, which had a moderate coefficient. For pwMS, there were moderate to excellent associations between BBT and the NHPT and FTT for both upper limbs. The correlations between hand grip strength and FFT were poor. This mobile app could be a useful and low-cost assessment tool in pwMS, allowing a simple evaluation and follow-up that has excellent correlation with clinical scales validated in this pathology.

## 1. Introduction

Multiple sclerosis (MS) is a chronic, inflammatory, demyelinating disease affecting the central nervous system (CNS) [1,2]. In MS, lesions occur in different focal areas of the CNS through the creation of demyelinating plaques with varying degrees of inflammation, gliosis, and neurodegeneration [3]. These alterations are associated with axon-neuronal loss and disruptions in nerve conduction, resulting in diminished or slowed signals, giving rise to the hallmark symptoms of this disease [4].

MS is the most common neurological condition leading to disability in young adults in Europe and North America. At present, the cause of this condition remains unknown, and it is thought to have a potential origin influenced by multiple factors [4]. MS is distinguished by a diverse array of symptoms and progression patterns. Specifically, upper limb (UL) impairments have a high prevalence in individuals with this condition [5]. According to Bertoni et al. [6], these UL alterations are present in approximately 60% of patients at the time of diagnosis and occur to a greater extent early in the course of the disease. The concerns most frequently mentioned by patients in relation to their UL are alterations in sensation, strength, and fine and gross motor skills. When cerebellar involvement occurs, dysdiadochokinesia (a common disorder in MS patients) may appear, which is characterized by a limitation or inability to perform rapid and alternating movements. Consequently, this results in a number of functional impairments, which may be combined with cognitive decline, impacting the ability to perform activities of daily living, which, in turn, directly affects the quality of life of people with MS (pwMS) [7,8,9].

The evaluation of the sensorimotor function of the hand has allowed the physical and cognitive assessment of numerous health conditions, as well as serving as a predictor of change and evolution [10]. Concretely, the finger tapping test (FTT) has been used to assess motor performance of the hand and fingers in healthy subjects [11] and a wide variety of pathologies, such as neurological disorders [12,13]. Further, the FTT is a tool developed as part of the Halstead Battery [14] for the neurophysiological assessment of motor control, being used in the evaluation of motor performance and eye–hand coordination [12,15]. The FTT has been used in the assessment of people with MS in several studies and has been found to be a reliable and valid assessment tool in this population to evaluate motor performance, as its scores are strongly associated with the estimated clinical severity of the disease [12,13].

mHealth is defined as the use of mobile devices to provide healthcare and information to consumers. Mobile applications (apps) are a promising tool in healthcare, offering new perspectives to patients and healthcare professionals, as well as to the general population. Apps focused on health, help to achieve a specific goal, or perform daily activities [16]. Since smartphones became popular, numerous health-focused apps have been developed [10]. The development of FTT applications has provided low-cost objective tools that have solved the difficulties presented by mechanical calibrators, which have the disadvantage of having to exert a certain force as well as exert the movement on a single axis of movement, which is limiting for certain groups of patients [10]. In recent years, an exponential increase in the use of mHealth in the development of everyday activities has been observed [17]. The interaction of users with these applications is generally simple, and the possibility of their application to various diseases has been studied [18]. Different mobile applications have been developed to assess UL coordination via mobile smartphones [18,19], as well as for self-management of pwMS [20]. However, the everyday use of smartphones has raised the question of their usefulness as a clinical tool. Prior to their clinical use, these devices and applications should be evaluated in terms of feasibility and psychometric properties, including construct validity (the relationship between the measure of interest and other related measures) and test–retest reliability. In this sense, several FTT applications have been designed, such as the one developed by SYBU (Data Digital Finger Tapping Test (version 3.5)) [21] or the Hand Assessment Test app [22], which are part of motor assessment batteries. Unlike these apps, the TappingPro^®^ mobile app [23] is specific in its analysis of FTT and provides specific and detailed information that can be useful in clinical and research settings. The TappingPro^®^ mobile app provides values that no other mobile app has presented: number of beats per unit of time, explosive speed (number of beats in the first 5 s), fatigue (comparison of beats per interval), and acceleration (time up to 60 beats).

The aim of the present study was to evaluate the intra- and inter-rater reliability of the TappingPro^®^ mobile app and its validity with validated clinical scales for motor performance, first in healthy subjects and secondarily with a representative sample of pwMS with a mild to moderate severity of the illness. The initial hypothesis of this study was that the TappingPro^®^ mobile app would present excellent intra- and inter-rater reliability and a correlation with other motor performance measures in pwMS.

## 2. Materials and Methods

### 2.1. Design

An observational study was conducted. This study followed the Helsinki Declaration and was approved by the Local Ethical Committee in Madrid (reference number: 100320229522). Informed consent was obtained from all participants prior to the start of the study. The STROBE (Strengthening the Reporting of Observational Studies in Epidemiology) guidelines were followed to standardize the reporting of this work [24].

### 2.2. Participants

#### 2.2.1. Healthy Subjects

The voluntary participation of healthy subjects was requested. Inclusion criteria for healthy subjects were as follows: (1) age between 18 and 60 years; (2) absence of cognitive impairment, with ability to understand instructions and a score equal to or greater than 24 on the Minimental Test [25]. The exclusion criteria were as follows: (1) diagnosis of any disease or condition that may interfere with this study; (2) use of medications that may influence neuromuscular function (muscle relaxants or some analgesics, among others); (3) presence of surgeries on the upper limb in the 6 months prior to the start of the study; (4) presence of musculoskeletal restrictions that may interfere with the performance of the test; (5) the presence of visual disorders noncorrected by optical devices; (6) use of caffeine, tea, energy drinks, or supplements during the 24 h before each study visit.

#### 2.2.2. MS Subjects

The recruitment of pwMS was carried out by sending information about the study to different patient associations in Comunidad de Madrid (Spain), with the pwMS themselves being the ones who contacted the researchers to participate in the study.

The inclusion criteria for pwMS were as follows: (1) age between 18 and 60 years; (2) confirmed diagnosis of MS through the McDonald criteria [26], with an evolution time of more than two years; (3) absence of cognitive impairment, with ability to understand instructions and score equal to or greater than 24 on the Minimental Test [25]; (4) score on the Expanded Disability Status Scale (EDSS) between 3.0 and 7.5 [27]; (5) stable medical treatment for at least six months prior to surgery; (6) score less than or equal to 4 points on the “Pyramidal Function” section of the EDSS functional scale; (7) upper extremity muscle tone no greater than 2 points on the modified Ashworth Scale [28]; (8) upper extremity muscle balance equal to or greater than 3 points. The exclusion criteria were as follows: (1) diagnosis of another neurological disease or musculoskeletal disorder other than MS; (2) having suffered an exacerbation or hospitalization in the last 3 months before starting the assessment protocol, nor during the therapeutic intervention process; (3) having received a course of intravenous or oral steroids, 6 months before the start of the assessment protocol and within the therapeutic intervention period; (4) presence of visual alterations not corrected by means of ocular devices; (5) use of medications that may influence neuromuscular function (muscle relaxants or analgesics, among others); (6) presence of cerebellar affectation according to medical history or symptomatology suggesting cerebellar affectation, such as the presence of dysdiacokinesias; (7) presence of surgeries on the upper limb in the 6 months prior to the start of the study; and (8) use of caffeine, tea, energy drinks, or supplements during the 24 h before each study visit.

### 2.3. Procedure

The recommendations of Zayas-García et al. [29] and previous studies on the clinical validation of apps in the assessment of different motor aspects [30,31,32] were followed to carry out the present investigation. First, the psychometric properties described above were studied in a population of healthy adults.

Subsequently, a representative sample of patients with MS was recruited, with an EDSS between 3.0 and 7.5, for the validation process of the app in this neurodegenerative disorder.

The procedure was the same regardless of the group of participants. 

#### 2.3.1. Finger Tapping Test

A 4.7″ cell phone, the iPhone SE (Apple, Inc., Los Altos, CA, USA), incorporating a 1.85 GHz 64-bit ARMv8-A processor (dual core) and 2 GB of RAM, was used to perform the FTT. This performance evaluates finger mobility in terms of manual dexterity. The objective of the exercise was to touch the screen with the second finger as many times as possible in 10 s, and in 1 min, first with one upper limb and then with the other. The FTT was performed with the TappingPro^®^ mobile app version 1.0.8.1. This app allows you to calculate the average value of taps in an interval of 5 s, the number of taps, and the duration of taps (ms), allowing its visualization in graphs that facilitate its use. In addition, indirect fatigue calculations can be obtained because the number of taps per time interval can be obtained.

The participants’ arms were placed in pronation, with elbow flexion of 40–45°. The experimenter explained the exercise to the participants, allowing one trial with each hand before the test was performed. The experimenter placed the cell phone on the table with the screen pointing upward at a distance comfortably reachable by the participant. To perform the test, the participant’s hand was relaxed and open, with the cell phone screen below the second finger, with which taps were made. The second finger performed the test in extension of the distal and proximal interphalangeal joints, with flexion/extension of the metatarsophalangeal joint. The experimenter initiated each exercise by pressing a “start” button and monitored its execution. The app starts the test with the first touch from each participant. There was a 1-minute rest interval between each test. If an adverse event occurred in the execution, the trial was discarded, and the exercise was repeated.

#### 2.3.2. Intra-Rater Reliability

For test reliability, the same procedure described for the finger tapping test was performed using the TappingPro^®^ mobile app on all participants at maximum speed for 10 s and 1 min on two different occasions, separated by 7 days, by the same tester and under identical conditions at the same time of day.

#### 2.3.3. Inter-Rater Reliability

To assess inter-rater reliability, the same procedure described for the finger tapping test was performed using the TappingPro^®^ mobile app on all participants at maximum speed for 10 s and 1 min by two different raters on the same day, 30 min apart, to avoid fatigue.

#### 2.3.4. Construct Validity

The aim of this research was to study the construct validity between the number of taps with the second finger in 10 s and 1 min, assessed by the TappingPro^®^ mobile app, and fine manual dexterity, assessed by validated clinical scales. The scales used were:

Hand grip strength

A Jamar^®^ hydraulic hand dynamometer (JLW Instruments Chicago, IL, USA) was employed to assess grip strength, providing precise and consistent measurements in both pounds and kilograms. Each patient completed three grip exercises, and the average values were documented. Data for both upper limbs were noted in kilograms. Widely recognized as one of the foremost objective instruments for assessing grip strength, the Jamar^®^ hydraulic hand dynamometer is esteemed for its reliability, sensitivity, and user-friendly design. It is highly recommended by the American Society of Hand Therapists and the Brazilian Society of Hand Therapists [33].

Box and Blocks Test (BBT)

This test measures gross manual dexterity in both ULs. The BBT consists of a 53.7 × 25.4 cm box divided into two spaces by a 15.2 cm high panel, in which 150 blocks are located. The test consisted of moving the maximum number of blocks from one compartment to the other, one at a time, for one minute with one UL [34]. The same procedure was then repeated with the other UL. At the end of each test, the examiner obtained the score by counting the number of blocks moved by the subject, following the standardized procedure [31]. The BBT is a quick, simple, and reliable measure of manual dexterity [35].

Nine Hole Peg Test (NHPT)

This test is used to measure fine manual dexterity. The test consists of recording the time required to insert nine pegs of 0.64 cm in diameter and 3.2 cm long in a square board with nine holes spaced 3.2 cm apart and then returning them to their place of origin. For this purpose, the subject began by performing the test with one UL and subsequently with the other UL [36,37].

Specific clinical testing procedures were applied to reduce procedural and interviewer test bias (Table 1).

### 2.4. Statistical Analysis

Statistical analysis was performed using SPSS 29.0 statistical software for Windows (SPSS Inc., Chicago, IL, USA, version 29.0). The Intraclass Correlation Coefficient (ICC) [38] will be used to assess intra- and inter-rater reliability. The ICC was estimated, and its 95% confidence intervals were calculated based on absolute agreement and a mixed-effects model (ICC 3.1). The ICC values were interpreted as excellent (>0.90), good (0.76–0.90), moderate (0.50–0.75), and low (<0.50) [39].

Absolute reliability will be defined by estimating the Standard Error of the Measurement (SEM), the Minimal Detectable Change (MDC), and the Standard Deviation of the differences between raters (SDdiff). The SEM and MDC will be calculated using the following equations: SEM = SDdiff × $\sqrt{1}$-ICC, and MDC = 1.96 × $\sqrt{2}$ × SEM [40]. To calculate MDC independent of the units of measurement, the MDC% was defined as (MDC/X) ∗ 100, where X is the mean for all observations from test sessions 1 and 2 [41].

A Bland–Altman analysis with 95% limits of agreement will be performed to assess the intra- and inter-rater reliability of the app. The bias and limits of agreement are shown in the plots of the recorded parameters. The mean score is plotted on the *x*-axis, and the difference between observers or sessions (mean of the differences) is plotted on the *y*-axis (mean of the difference ± 1.96 SD, standard deviation). The width of the limits of agreement and the distance of the mean of the differences from zero can be used to interpret the errors between measurements. Bland-Altman plots allow comparisons between evaluators or different sessions when evaluating the same data set to analyze the level of agreement [42]. The level of statistical significance was set at a *p*-value of less than 0.05 [43].

Pearson and Spearman correlation coefficients investigated the relationship between the TappingPro^®^ (number of beats) and the BBT, NHPT, hand grip strength, and caffeine consumption. Correlation coefficients of 0.00–0.49 were interpreted as poor, those of 0.50–0.79 as moderate, and those of 0.80 or higher as excellent [44].

### 2.5. Sample Size Calculation

The sample size was calculated according to the work of Walter et al. (1998) [45]. The sample size was determined using the ICC and the number of raters. Thus, a minimum acceptable ICC (p0) of 0.6 and an expected ICC (p1) of 0.8 were established. With these parameters and according to the contingency tables of Walter et al. [42], the required sample size was 39 subjects for the reliability analysis. On the other hand, the sample size for the validity study was calculated. The G*Power software (version 3.1.9.2) was used for this purpose. The following sample size parameters were established using Pearson’s correlation coefficient: two-tailed, an alpha error of 0.05, and a power of 0.95, resulting in a required sample size of 38 participants. The final sample size selected was 42 subjects, considering a possible loss of 10%.

## 3. Results

**Figure 1:**
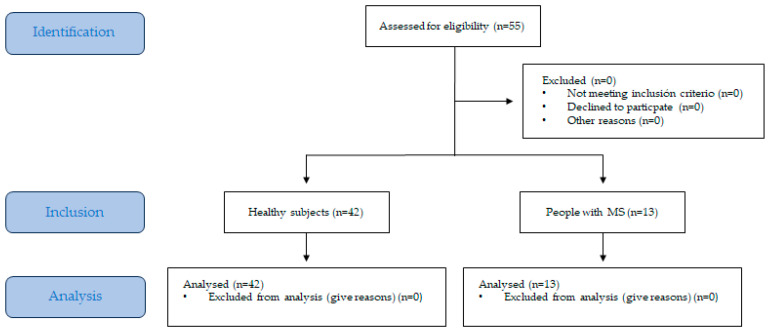
Flow chart.

The sample of healthy subjects consisted of 42 subjects, 25 of whom were female (59.5%), 37 of the participants were right-handed (88.1%), 3 were ambidextrous (7.1%), and 2 were left-handed (4.8%). The sample of pwMS consisted of 13 subjects, 5 of whom were female (38.46%); 10 of the pwMS had more impairment of the left UL (76.92%); and 3 had more impairment of the right UL (23.08%). The flow chart is shown in Figure 1. The remaining anthropometric data and clinical scale scores are shown in Table 2.

### 3.1. Intra-Rater Reliability

The intra-rater reliability showed a good correlation for both upper limbs and both FTT for healthy subjects, and an excellent correlation for upper limbs and both FTT for pwMS (Table 3).

**Figure 2:**
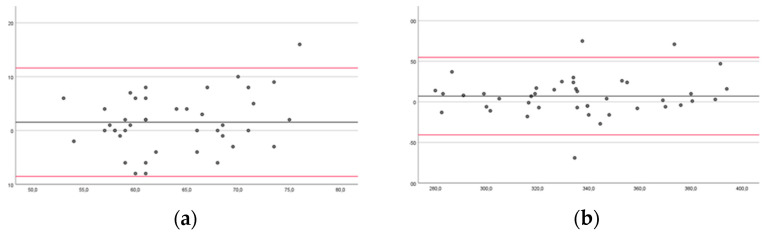

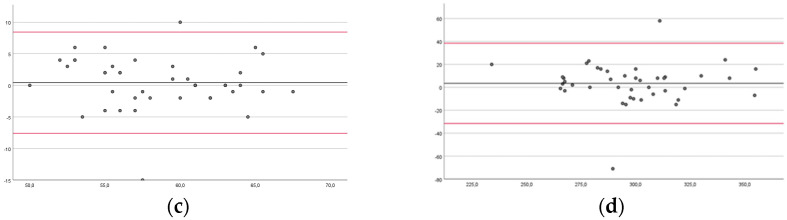
Bland–Altman plots of healthy subjects comparing results between sessions of measurements (for the Finger Tapping Test (FFT)) for the dominant upper limb during 10 (**a**) and 60 s (**b**) and for the non-dominant upper limb during 10 (**c**) and 60 s (**d**). The mean score is plotted on the *x*-axis, and the difference between observers (mean of the differences) is plotted on the *y*-axis (mean difference ± 1.96 SD).

In the Bland–Altman plots, the limit for agreement for healthy subjects for dominant UL (DUL) in FTT 10 s and 60 s were −8.52 to 11.62 and −40.59 to 54.88; for non-dominant UL (NDUL) in FTT 10 s and 60 s were from −7.59 to 8.45 and from −31.5 to 38.5, respectively (Figure 2).

**Figure 3:**
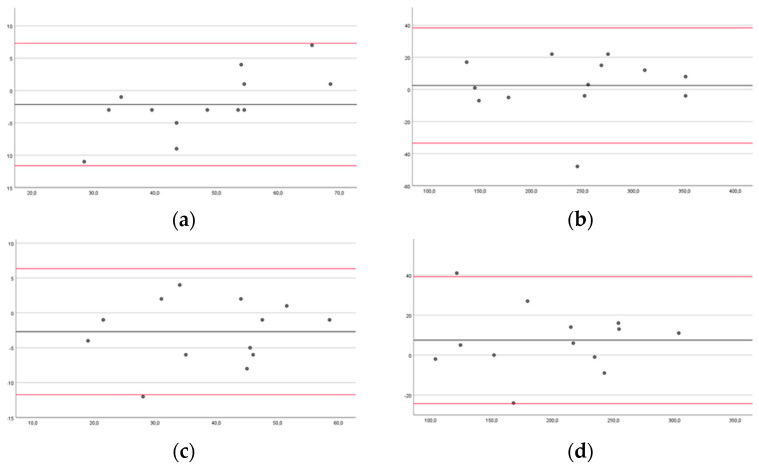
Bland–Altman plots for people with multiple sclerosis comparing results between sessions of measurements for the Finger Tapping Test (FFT) for the less-affected upper limb during 10 (**a**) and 60 s (**b**) and for the more-affected upper limb during 10 (**c**) and 60 s (**d**). The mean score is plotted on the *x*-axis, and the difference between observers (mean of the differences) is plotted on the *y*-axis (mean difference ± 1.96 SD).

For pwMS, the less-affected UL (LAUL) in FTT 10 s and 60 s were from −11.62 to 7.31 and −33.46 to 38.39, respectively; the more-affected UL (MAUL) in FTT 10 s and 60 s were from −11.62 to 7.31 and −24.3 to 39.22, respectively (Figure 3).

### 3.2. Inter-Rater Reliability

The ICC for the inter-rater reliability was good for the left hand in the FTT 10 s of the healthy subjects and excellent for all the other measurements of the healthy subjects and pwMS (Table 4).

**Figure 4:**
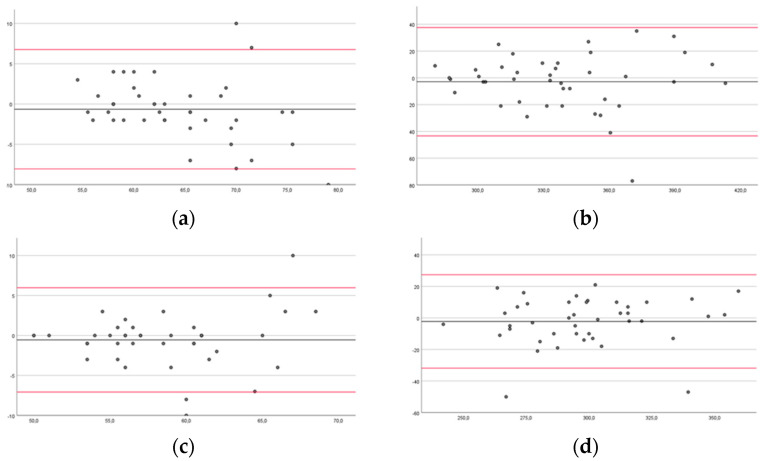
Bland–Altman plots of healthy subjects comparing results between raters for the Finger Tapping Test (FFT) for the dominant upper limb during 10 (**a**) and 60 s (**b**) and for the non-dominant upper limb during 10 (**c**) and 60 s (**d**). The mean score is plotted on the *x*-axis, and the difference between observers (mean of the differences) is plotted on the *y*-axis (mean difference ± 1.96 SD).

In the Bland–Altman plots, the limits for agreement for healthy subjects for DUL in FTT 10 s and 60 s were from −8.05 to 6.76 and −43.32 to 37.61, respectively; for NDUL in FTT 10 s and 60 s, they were from −7.07 to 5.98 and −31.84 to 27.41, respectively (Figure 4).

**Figure 5:**
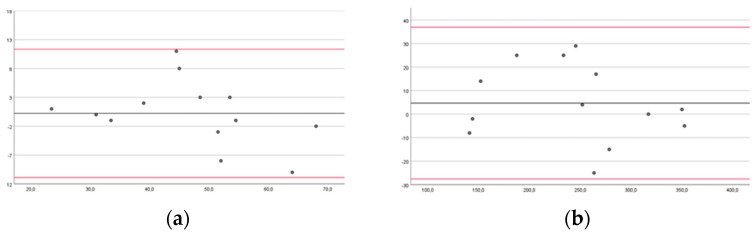

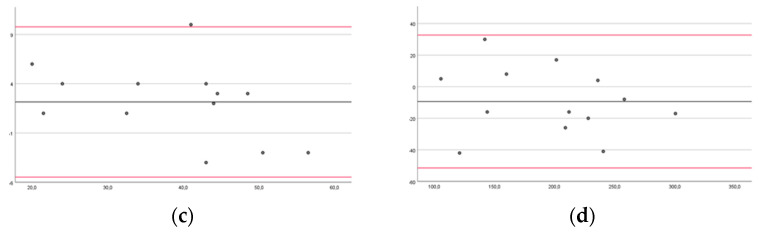
Bland–Altman plots of people with multiple sclerosis comparing results between sessions of measurements for the Finger Tapping Test (FFT) for the less-affected upper limb during 10 (**a**) and 60 s (**b**) and for the more-affected upper limb during 10 (**c**) and 60 s (**d**). The mean score is plotted on the *x*-axis, and the difference between observers (mean of the differences) is plotted on the *y*-axis (mean difference ± 1.96 SD).

For pwMS, LAUL in FTT 10 s and 60 s were from −10.89 to 11.35 and −27.58 to 36.96, respectively; MAUL in FTT 10 s and 60 s were from −5.47 to 9.78 and −51.48 to 32.71, respectively (Figure 5).

### 3.3. Construct Validity

The variables for which Spearman was performed because it did not follow a normal distribution in the healthy subjects were FTT 10 s in both UL and FTT 60 s in DUL. In pwMS, the variables that did not follow a normal distribution were NHPT in both ULs. Pearson was used for the rest of the variables, both in healthy subjects and pwMS, since it followed a normal distribution.

The correlation analysis for healthy subjects showed poor associations between all variables, except for the association between hand grip strength and the FTT 60 s in NDUL, which had a moderate coefficient (Table 5). The correlation analysis for pwMS showed excellent associations between BBT and all the measures of the FTT and between the NHPT and FTT 10 s for LAUL and FTT 60 s for MAUL. The correlations between NHPT and FTT 60 s for MAUL and FTT 10 s for LAUL were moderate. The correlations between hand grip strength and FTT were poor (Table 6).

## 4. Discussion

The purpose of the present study was to evaluate the intra- and inter-rater reliability of the TappingPro^®^ mobile app and the construct validity between this app and validated clinical scales for motor performance in healthy subjects and pwMS.

Our findings showed good intra-rater reliability in healthy subjects and excellent reliability in pwMS. The inter-rater reliability was good for the left hand in the FTT 10 s of the healthy subjects, and excellent for all the other measurements of the healthy subjects and pwMS. However, the Bland–Altman plots may be more useful than the ICC, as they can be readily and easily interpreted in a meaningful way in both clinical and research settings. Specifically, the width of the limits of agreement is useful in understanding the level of agreement or disagreement between observers, measurements, or systems [42,46]. In the intra-rater reliability, the range of the limits of agreement was slightly narrower for healthy subjects for NDUL in FTT 10 s (−7.59 to 8.45) and 60 s (31.5 to 38.5), and for pwMS for MAUL in FTT 10 s (−11.62 to 7.31) and 60 s (−24.3 to 39.22). There was no outlier for LAUL in FTT 10 s for pwMS, and at most three outliers for DUL in FTT 60 s for healthy subjects. In the inter-rater reliability, the range of the limits of agreement was slightly narrower for healthy subjects for NDUL in FTT 10 s (−7.07 to 5.98) and 60 s (−31.84 to 27.41), and for pwMS for MAUL in FTT 10 s (−5.47 to 9.78) and for LAUL in FTT 60 s (−27.58 to 36.96). There was no outlier for LAUL in FTT 10 s and 60 s and for MAUL in FTT 60 s for pwMS, and at most three outliers for DUL and NDUL in FTT 10 s for healthy subjects.

Regarding the construct validity of the app with validated clinical scales for motor performance in healthy subjects and pwMS, correlation analysis for healthy subjects showed poor correlations between the 10 and 60 s FTT but excellent correlations between these two tests and the manual dexterity tests (BBT and 9 HPT). The correlation analysis for pwMS showed excellent associations between BBT and all the measures of the FTT and between the NHPT and FTT 10 s for LAUL and FTT 60 s for MAUL. The correlations between NHPT and FFT 60 s for MAUL and FFT 10 s for LAUL were moderate. The correlations between hand grip strength and FFT were poor. These findings could be interpreted as the mobile application would present a construct validity with validated clinical scales for motor performance in pwMS related to coordination outcomes (BBT and NHPT), considered a convergent construct validity, but not with hand grip strength.

It is noteworthy to mention that mobile applications have become one of the most widely used tools in healthcare contexts, owing to their capacity to facilitate individual care, provide seamless access to information and communication, and enable the monitoring of health-related parameters [16,18]. Furthermore, numerous studies have sought to investigate and validate their use in pwMS to complement their medical and rehabilitative treatment, promote engagement in physical and mental activities, and facilitate medication monitoring [47,48,49,50]. For example, Pedullà et al. [47], attempted to verify the effectiveness of a mobile application called COGNI-TRAcK for intensive and adaptive treatment based on memory exercises aimed at improving the cognitive state of pwMS. Golan et al. [48] sought to assess the utility and validity of using a mobile application (MyMS&Me) based on an electronic diary to evaluate adherence and the effectiveness of pharmacological regimens in this population. Finally, Nasseri et al. [49] and Van Geel et al. [50] aimed to understand the effects of using two mobile applications on motivation to engage in physical activity, fatigue, and cognitive levels in pwMS. However, few studies have investigated the validity and reliability of applications designed to assess UL motor function in people with neurological disorders. As an example, the study conducted by Mollà-Casanova et al. [51] aimed to determine the validity and reliability of a mobile application (the Hand Assessment Test) for assessing UL function in stroke individuals. The findings of this research supported the efficacy of this tool in fulfilling the required function, thus establishing it as a complementary instrument for assessing manual function in individuals with stroke.

To our best knowledge, applications related to the assessment of motor function, specifically UL motor dexterity, are scarcely explored in pwMS. In this context, the Tapping Pro^®^ mobile application, despite not being originally designed for the purpose of evaluating UL motor dexterity in individuals with MS, might offer the possibility of recording a set of parameters that may provide insights at the clinical level. According to the results obtained in the present study, with excellent intra- and inter-observer reliability and strong correlation with motor dexterity scales (BBT and NHPT), we could assume that the FTT within the Tapping Pro^®^ application emerges as a reliability tool for assessing manual dexterity and with a convergent construct validity in pwMS. Furthermore, its accessibility, ease of use, ability to gather quantitative objective data, portability, and low cost (1.99 euros) are among the inherent benefits of using this application for evaluating UL motor dexterity in pwMS [29].

It is important to mention that the individuals with MS included in this study exhibited an average age of 51.69 (±6 years). According to Mathiowetz et al. [34], the average scores for healthy individuals aged between 45 and 57 years on the BBT range from 73.6 to 83 points, varying depending on the limb assessed, the subject’s gender, and their age. However, the scores obtained in this study in subjects with MS were 49.62 (±18.36) and 56.46 (±17.53) in the MAUL and LAUL, respectively. Furthermore, the average values of the NHPT test for healthy subjects show a range from 17.3 to 21.0 s [52], whereas individuals with MS included in our study exhibited results ranging from 33.63 (±22.31) to 56.01 (±49) seconds. Comparing the standardized average data of healthy subjects [34,52] to those with MS included in this study, it is readily apparent that the disease has a significant impact on both gross and fine motor skills in pwMS, as well as on UL coordination. Additionally, it is well known that loss of manual dexterity is associated with a decrease in independence to perform activities of daily living and, consequently, a decline in quality of life in pwMS [5,6,9]. Therefore, the use of valid, reliable, user-friendly, and cost-effective tools, such as the FTT within the Tapping Pro^®^, could become an interesting technological tool for monitoring manual dexterity and coordination to detect and/or treat their early deterioration in pwMS, owing to its close correlation (convergent construct validity) with outcomes, such as the BBT and the NHPT. So, future studies could be conducted in this line to corroborate these hypotheses.

There are several limitations to this study that warrant attention. Firstly, it is important to note that the findings may not be applicable to the broader population of individuals with MS or other neurological disorders, given that the study focused exclusively on patients scoring between 3.0 and 7.5 on the EDSS scale. Secondly, the sampling technique utilized might have introduced selection bias, considering that participants were sourced solely from a single MS association in a particular geographical area. Third, the Tapping Pro^®^ app is only available for the iOS operating system, which may restrict its usage in situations where such devices are not available. Fourth, the Tapping Pro^®^ app costs less than USD 2, but having a device with an iOS operating system, such as the one used in this study, costs more than USD 400/370 Euros, which could be socioeconomically challenging and most likely unavailable in some areas of the world.

Future studies could use our protocol to monitor disease progression, therapeutic effects, and/or for the early detection of motor symptoms in pwMS.

## 5. Conclusions

The Tapping Pro^®^ app, available for the iOS operating system, showed good intra-rater reliability in healthy subjects and excellent reliability in pwMS. The inter-rater reliability was good for the left hand in the FTT 10 s of the healthy subjects and excellent for all the other measurements of the healthy subjects and pwMS. Correlation analysis for healthy subjects showed poor associations between all variables, and the ICCs between FTT and hand grip strength were poor for both populations. Correlation coefficients were moderate to excellent with coordination and dexterity assessments, except for correlations with hand grip strength, which were poor. So, this mobile application presents convergent construct validity with the validated clinical scales for coordination in pwMS, but not with hand grip strength. These findings could be interpreted as the mobile application would present a construct validity with validated clinical scales for motor performance in pwMS related to coordination outcomes (BBT and NHPT), considered a convergent construct validity, but not with hand grip strength.

## Figures and Tables

**Table 1:** Clinical procedures for motor skill assessments.

1—All tests were conducted in a quiet, enclosed room to reduce the effects of visual and auditory interference.
2—The subjects were tested in the same room, with the same conditions, the same chair, and the same table.
3—The subjects were instructed to perform the test in the same specific posture for each test.
4—The tests were performed in the same order and with the same time interval.
5—Subjects were not allowed to wear jewelry, watches, or other accessories on the upper extremities.
6—All subjects were allowed to perform a pretest for each test.

**Table 2:** Sociodemographic data and scale scores of healthy subjects and people with multiple sclerosis.

	Healthy Subjects (*n* = 42)	People with MS (*n* = 13)
Age	25.05 (7.58)	51.69 (6)
Height	170.6 (9.02)	173.5 (2.54)
Weight	68.83 (11.38)	70.2 (5.59)
EDSS Median [IR]	-	6.5 [3]
Jamar DUL/LAUP	34.26 (10.35)	33.7 (12.14)
Jamar NDUL/MAUP	32.19 (10.07)	30.26 (12.64)
BBT DUL/LAUP	68.14 (8.26)	56.46 (17.53)
BBT NDUL/MAUP	65.12 (7.08)	49.62 (18.36)
NHPT DUL/LAUP	18.13 (2.1)	56.01 (49)
NHPT NDUL/MAUP	19.36 (1.78)	33.63 (22.31)
FTT DUL/LAUP 10 s	64.27 (6.25)	46.81 (12.77)
FTT NDUL/MAUP 10 s	58.44 (5.04)	38.69 (11.45)
FTT DUL/LAUP 60 s	338.54 (34.22)	244.81 (71.38)
FTT NDUL/MAUP 60 s	297.48 (26.68)	196.77 (58.115)

BBT, Box and Blocks Test; DUL, dominant upper limb; FFT, Finger Tapping Test; IR, interquartile range; LAUL, less-affected upper limb; MAUL, more affected upper limb; NDUL, non-dominant upper limb; and NHPT, Nine Hole Peg Test.

**Table 3:** Intra-rater reliability of the Finger Tapping Test for healthy subjects and people with multiple sclerosis.

	Number of Taps			Intra-Rater Reliability		
Healthy subjects	Session 1	Session 2	MD	ICC	95% CI	*p*-Value
DUL 10 s	64.5 (7.06)	62.95 (5.78)	1.55 (5.14)	0.882	0.63 to 0.894	<0.001
NDUL 10 s	58.83 (4.69)	58.4 (5.03)	0.43 (4.09)	0.787	0.604 to 0.885	<0.001
DUL 60 s	339.02 (34.99)	331.88 (32.13)	7.14 (24.36)	0.841	0.702 to 0.915	<0.001
NDUL 60 s	299.52 (26.94)	296.02 (27.12)	3.5 (17.85)	0.876	0.771 to 0.933	<0.001
People with MS	Session 1	Session 2	MD	ICC	95% CI	*p*-Value
LAUL 10 s	46.7 (14.01)	48.85 (10.68)	−2.15 (4.83)	0.956	0.856 to 0.987	<0.001
MAUL 10 s	37.62 (12.4)	40.31 (11.79)	−2.69 (4.61)	0.953	0.821 to 0.986	<0.001
LAUL 60 s	242.46 (74.44)	240 (73.46)	2.46 (18.33)	0.985	0.953 to 0.995	<0.001
MAUL 60 s	201.46 (61.44)	194 (61.93)	7.46 (16.21)	0.98	0.933 to 0.994	<0.001

CI, Confidence Internal; DUL, dominant upper limb; ICC, Intraclass Correlation Coefficient; LAUL, less-affected upper limb; MAUL, more affected upper limb; MD, Mean Difference; MS, multiple sclerosis; and NDUL, non-dominant upper limb.

**Table 4:** Inter-rater reliability of the Finger Tapping Test for healthy subjects and people with multiple sclerosis.

	Number of Taps			Inter-Rater Reliability		
Healthy subjects	Rater 1	Rater 2	MD	ICC	95% CI	*p*-Value
DUL 10 s	64.5 (7.06)	63.86 (5.82)	0.64 (3.78)	0.906	0.827 to 0.95	<0.001
NDUL 10 s	58.83 (4.69)	58.29 (5.11)	0.55 (3.33)	0.869	0.758 to 0.93	<0.001
DUL 60 s	339.02 (34.99)	336.17 (34.61)	2.86 (20.65)	0.904	0.822 to 0.948	<0.001
NDUL 60 s	299.52 (26.94)	297.31 (28.47)	2.21 (15.11)	0.920	0.852 to 0.957	<0.001
People with MS	Rater 1	Rater 2	MD	ICC	95% CI	*p*-Value
LAUL 10 s	46.69 (14.01)	46.92 (11.97)	−0.23 (5.67)	0.954	0.848 to 0.986	<0.001
MAUL 10 s	37.62 (12.4)	39.77 (10.81)	−2.15 (3.89)	0.965	0.87 to 0.99	<0.001
LAUL 60 s	242.46 (74.44)	247.15 (71.14)	−4.69 (16.46)	0.987	0.959 to 0.996	<0.001
MAUL 60 s	201.46 (61.44)	192.08 (56.62)	9.38 (21.48)	0.962	0.875 to 0.988	<0.001

CI, Confidence Internal; DUL, dominant upper limb; ICC, Intraclass Correlation Coefficient; LAUL, less-affected upper limb; MAUL, more affected upper limb; MD, Mean Difference; MS, multiple sclerosis; and NDUL, non-dominant upper limb.

**Table 5:** Healthy subjects’ correlations.

	**JAMAR DUL**			**BBT DUL**			**NHPT DUL**			**Coffee Consumption**		
	r/rho	CI	*p*-Value	r/rho	CI	*p*-Value	r/rho	CI	*p*-Value	r/rho	CI	*p*-Value
FTT 10 s DUL	0.369	0.073 to 0.605	0.016	0.444	0.162 to 0.659	0.003 *	−0.317	−0.566 to −0.014	0.041	0.098	−0.212 to 0.39	0.539
FTT 60 s DUL	0.354	0.056 to 0.594	0.022	0.452	0.172 to 0.665	0.003 *	−0.349	−0.59 to −0.05	0.023	−0.004	−0.308 to 0.3	0.98
	**JAMAR NDUL**			**BBT NDUL**			**NHPT NDUL**			**Coffee Consumption**		
	r/rho	CI	*p*-Value	r/rho	CI	*p*-Value	r/rho	CI	*p*-Value	r/rho	CI	*p*-Value
FTT 10 s NDUL	0.392	0.01 to 0.622	0.011	0.26	−0.048 to 0.523	0.096	0.087	−0.223 to 0.381	0.584	0.148	−0.163 to 0.432	0.349
FTT 60 s NDUL	0.526	0.264 to 0.716	<0.001*	0.287	−0.019 to 0.544	0.066	0.023	−0.283 to 0.325	0.884	−0.044	−0.343 to 0.263	0.78

BBT, Box and Blocks Test; CI, Confidence Internal; DUL, dominant upper limb; FTT, Finger Tapping Test; NDUP, non-dominant upper limb; and NHPT, Nine Hole Peg Test. * *p* < 0.008 is considered significant by the Bonferroni correction.

**Table 6:** People with multiple sclerosis correlations.

	**JAMAR LAUL**			**BBT LAUL**			**NHPT LAUL**		
	r/rho	CI	*p*-Value	r/rho	CI	*p*-Value	r/rho	CI	*p*-Value
FTT 10 s LAUL	0.402	−0.191 to 0.78	0.173	0.921	0.751 to 0.976	<0.001 *	−0.845	−0.953 to −0.55	<0.001 *
FTT 60 s LAUL	0.45	−0.134 to 0.802	0.123	0.958	0.862 to 0.988	<0.001 *	−0.72	−0.91 to −0.28	0.006 *
	**JAMAR MAUL**			**BBT MADUL**			**NHPT MADUL**		
	r/rho	CI	*p*-Value	r/rho	CI	*p*-Value	r/rho	CI	*p*-Value
FTT 10 s MAUL	0.373	−0.224 to 0.766	0.21	0.825	0.0502 to 0.945	<0.001 *	−0.785	−0.933 to −0.412	0.001 *
FTT 60 s MAUL	0.312	−0.289 to 0.736	0.299	0.0883	0.647 to 0.965	<0.001 *	−0.857	−0.956 to −0.58	<0.001 *

BBT, Box and Blocks Test; CI, Confidence Internal; FTT, Finger Tapping Test; LAUP, less-affected upper limb; MAUL, more-affected upper limb; and NHPT, Nine Hole Peg Test. * *p* < 0.008 is considered significant by the Bonferroni correction.

## Data Availability

The original contributions presented in the study are included in the article.

## Abbreviations

BBT	Box and Blocks Test
CI	Confidence Interval
CNS	Central Nervous System
DUL	Dominant upper limb
EDSS	Expanded Disability Status Scale
FTT	Finger Tapping Test
ICC	Intraclass Correlation Coefficient
LAUL	Less-affected upper limb
MAUL	More-affected upper limb
MDC	Minimal Detectable Change
MS	Multiple sclerosis
NDUL	Non-dominant upper limb
pwMS	People with multiple sclerosis
SD	Standard Deviation
SDdiff	Standard Deviation of the differences
SEM	Standard Error of the Measurement
UL	Upper limb
